# Supplementary material for: Independent tuning of outer membrane fluidity and mechanics in Gram-negative bacteria
Source: bioRxiv. 2026 Sep 19:2026.09.17.752130. Preprint. [Version 1] doi: 10.64898/2026.09.17.752130 (PMC13596451; doi:10.64898/2026.09.17.752130)
Supplement: Supplementary file 1 [file NIHPP2026.09.17.752130v1-supplement-1.pdf]

bioRxiv preprint doi: https://doi.org/10.64898/2026.09.17.752130; this version posted September 19, 2026. The copyright holder for this preprint (which was not certified by peer review) is the author/funder, who has granted bioRxiv a license to display the preprint in perpetuity. It is made available under a CC-BY 4.0 International license.

Supplementary Figures

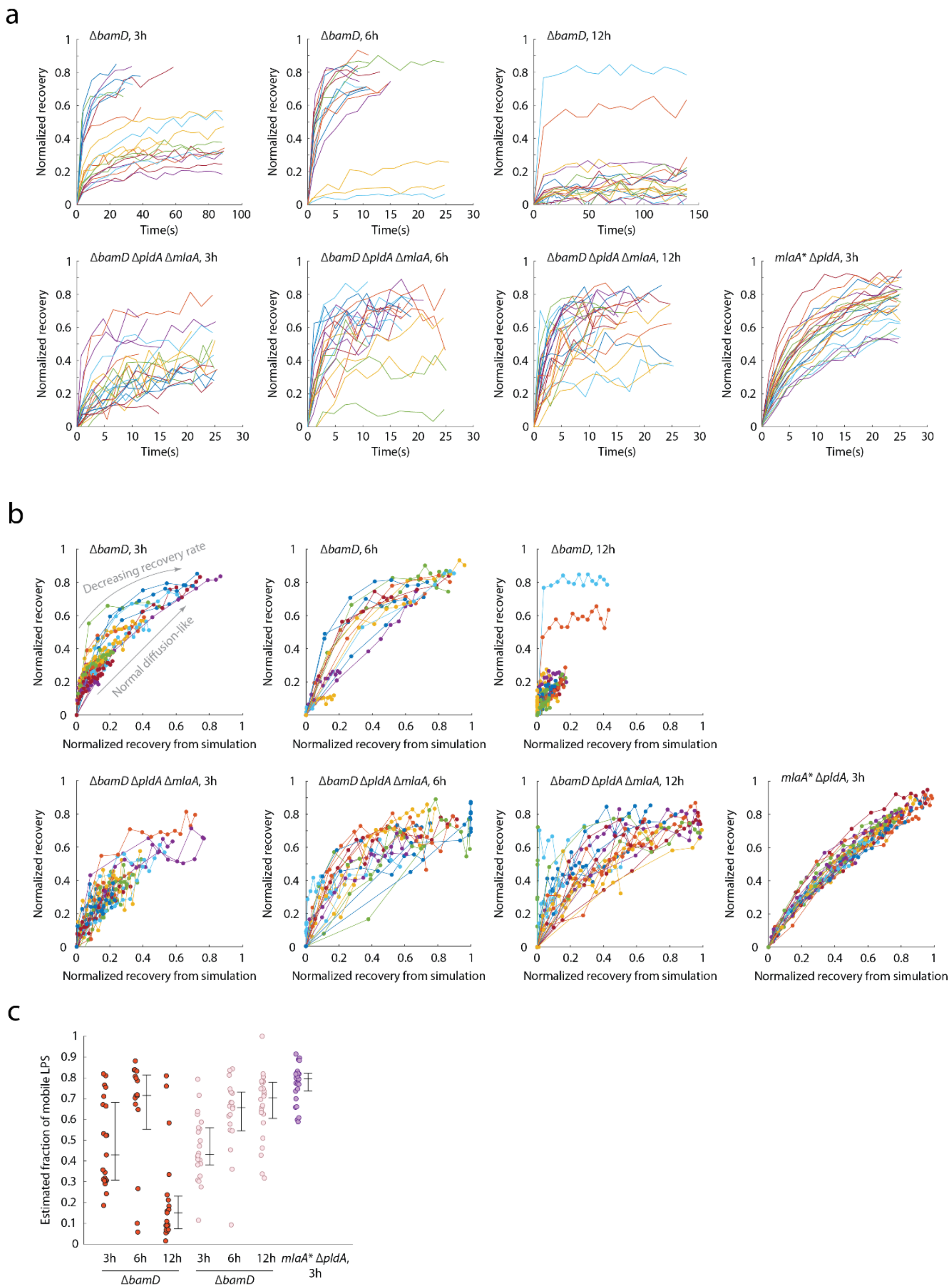

Figure S1: Fluorescence recovery of labeled LPS largely resembles normal diffusion in mutants with increased PL content in the OM outer leaflet. Across these conditions, recovery kinetics were broadly consistent with normal diffusion along the cell's long

bioRxiv preprint doi: https://doi.org/10.64898/2026.09.17.752130; this version posted September 19, 2026. The copyright holder for this preprint (which was not certified by peer review) is the author/funder, who has granted bioRxiv a license to display the preprint in perpetuity. It is made available under a CC-BY 4.0 International license.

axis (**Methods**), with minor deviations arising from a gradual decrease in recovery rate in some cells, suggesting subpopulations of more confined LPS molecules. Nonetheless, this confinement did not halt LPS motion, as indicated by the low estimated fraction of immobile LPS (**Methods**). Although ensemble FRAP measurements do not necessarily reflect Brownian motion at the molecular scale, the limited deviation from behavior predicted by linear diffusion suggests that PL insertion globally mobilizes LPS across the OM.

a) Normalized fluorescence recovery (**Methods**) of individual cells with reduced OM protein levels and/or increased PL content, measured across genetic perturbations and growth phases. Δ*bamD* (3 and 6 h), Δ*bamD* Δ*pldA* Δ*mlaA* (3, 6, and 12 h), and *mlaA** Δ*pldA* (3 h) cells exhibit rapid fluorescence recovery after photobleaching. Values of 0 and 1 represent the start and long-timescale limit of recovery, respectively, with t=0 marking the time point immediately after photobleaching.

b) Comparison between simulated and experimental normalized recovery. Δ*bamD* (3 and 6 h), Δ*bamD* Δ*pldA* Δ*mlaA* (3, 6, and 12 h), and *mlaA** Δ*pldA* (3 h) cells exhibit only minor deviations from the line y=x, indicating recovery kinetics resembling normal diffusion.

c) Mutant cells with high OM fluidity also exhibit low fractions of immobile LPS, as estimated from the normalized recovery curves (**Methods**). Center lines indicate medians, and error bars indicate the 25th and 75th percentiles.

bioRxiv preprint doi: https://doi.org/10.64898/2026.09.17.752130; this version posted September 19, 2026. The copyright holder for this preprint (which was not certified by peer review) is the author/funder, who has granted bioRxiv a license to display the preprint in perpetuity. It is made available under a CC-BY 4.0 International license.

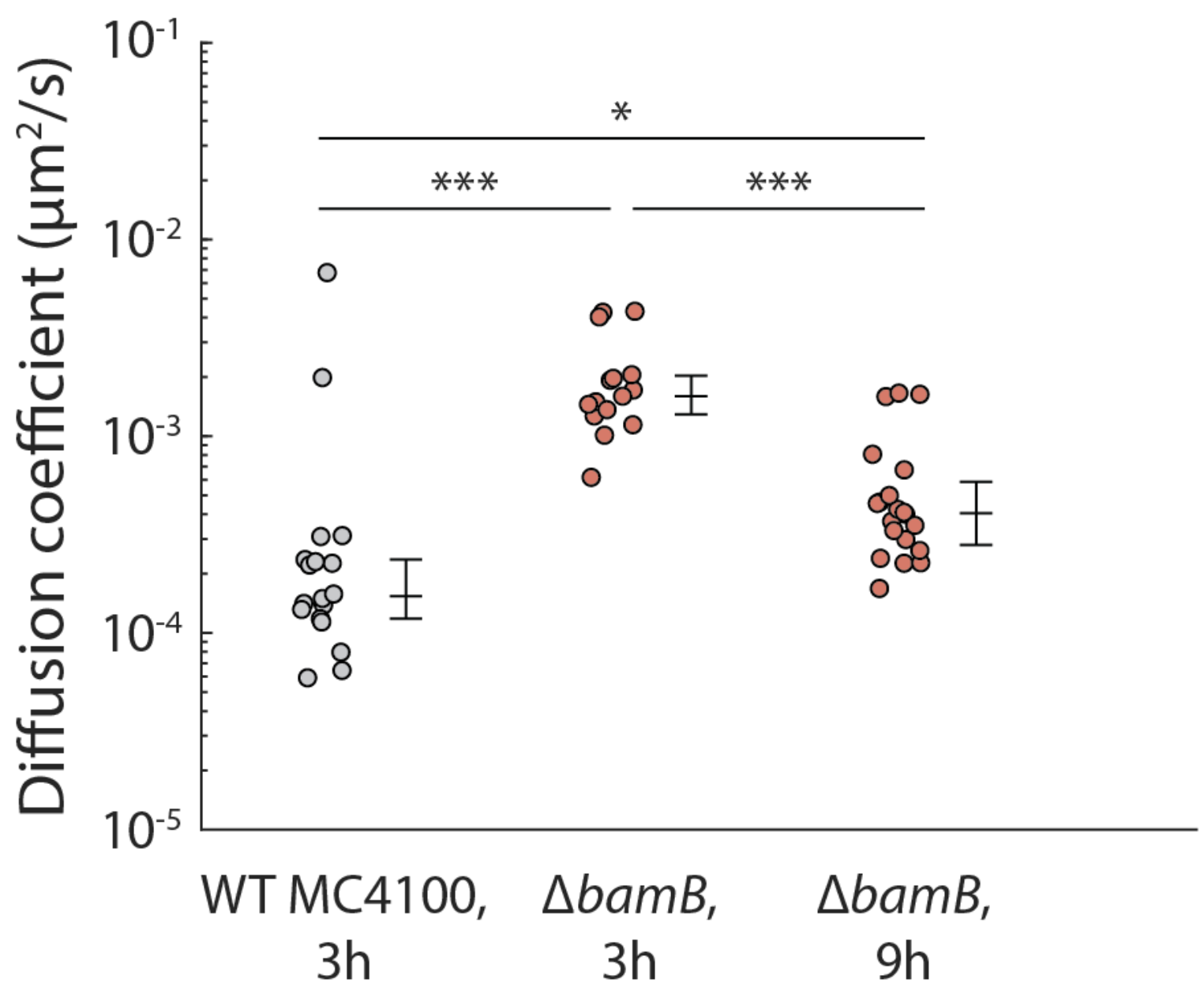

Figure S2: Insertion of PLs into the outer leaflet in Δ*bamB* cells increases OM fluidity. The increase in fluidity observed in Δ*bamB* cells grown in LB was growth-phase dependent and was lower than that observed in Δ*bamD* cells (Fig. 1b). Center lines indicate medians, and error bars indicate the 25th and 75th percentiles. $N > 14$ cells per condition. p values were calculated using Welch's t-test on $\log_{10}$-transformed diffusion coefficients. ***: $p < 0.001$; *: $p < 0.05$.

bioRxiv preprint doi: https://doi.org/10.64898/2026.09.17.752130; this version posted September 19, 2026. The copyright holder for this preprint (which was not certified by peer review) is the author/funder, who has granted bioRxiv a license to display the preprint in perpetuity. It is made available under a CC-BY 4.0 International license.

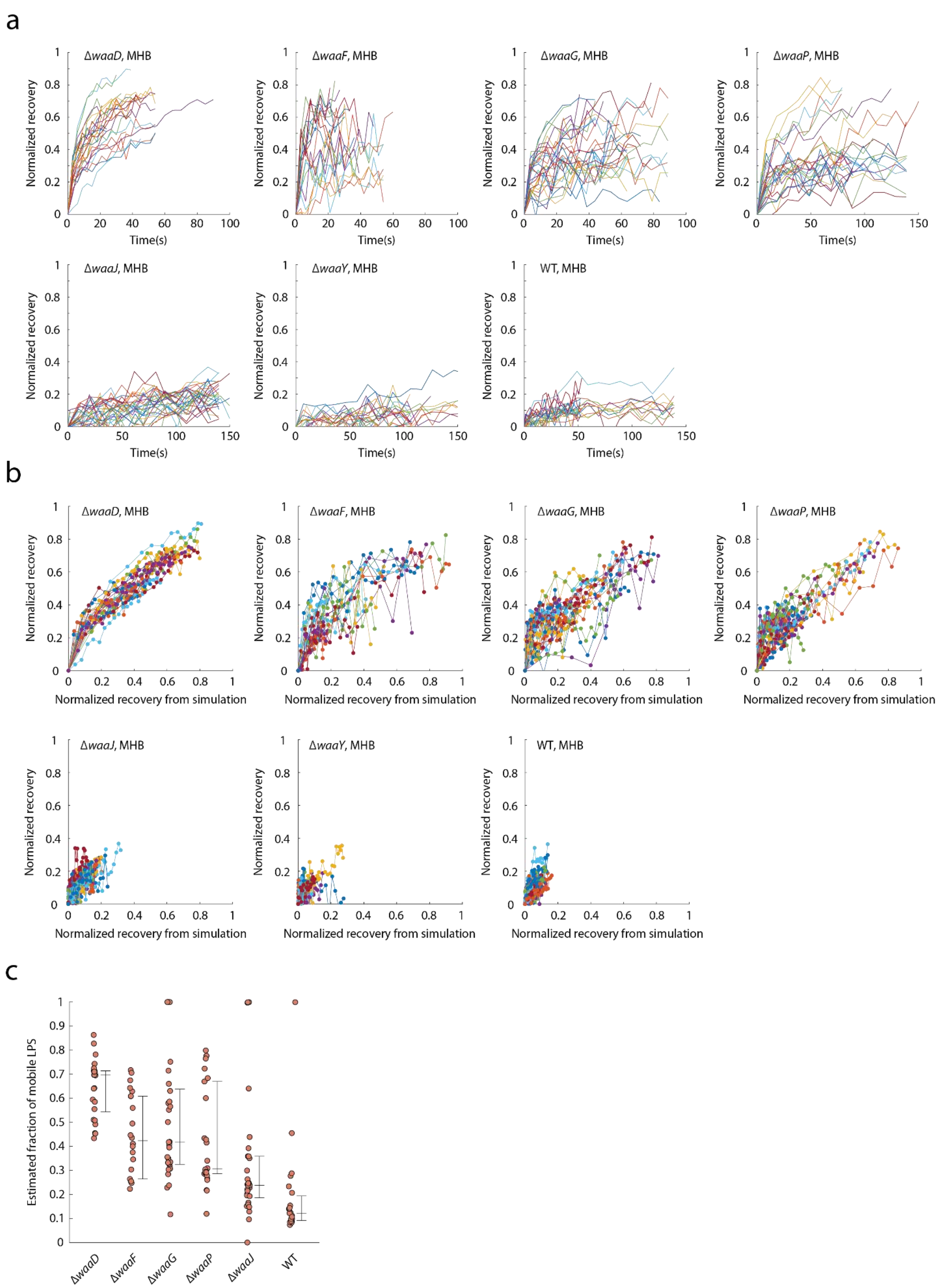

Figure S3: LPS core truncation increases OM fluidity and alters fluorescence recovery dynamics.

bioRxiv preprint doi: https://doi.org/10.64898/2026.09.17.752130; this version posted September 19, 2026. The copyright holder for this preprint (which was not certified by peer review) is the author/funder, who has granted bioRxiv a license to display the preprint in perpetuity. It is made available under a CC-BY 4.0 International license.

a) Normalized fluorescence recovery curves (**Methods**) for individual *waa* mutant cells with truncated LPS. Values of 0 and 1 represent the start and long-timescale limit of recovery, respectively, with t=0 corresponding to the time point immediately after photobleaching.

b) Comparison between experimentally measured fluorescence recovery and simulated recovery assuming normal diffusion. Mutants with truncated LPS show recovery dynamics that largely follow the y=x relationship across *waa* deletions.

c) Fraction of mobile LPS as a function of the remaining length of the core oligosaccharide. Shorter LPS cores are associated with higher OM fluidity and an increased mobile fraction. Center lines indicate medians, and error bars indicate the 25th and 75th percentiles.

bioRxiv preprint doi: https://doi.org/10.64898/2026.09.17.752130; this version posted September 19, 2026. The copyright holder for this preprint (which was not certified by peer review) is the author/funder, who has granted bioRxiv a license to display the preprint in perpetuity. It is made available under a CC-BY 4.0 International license.

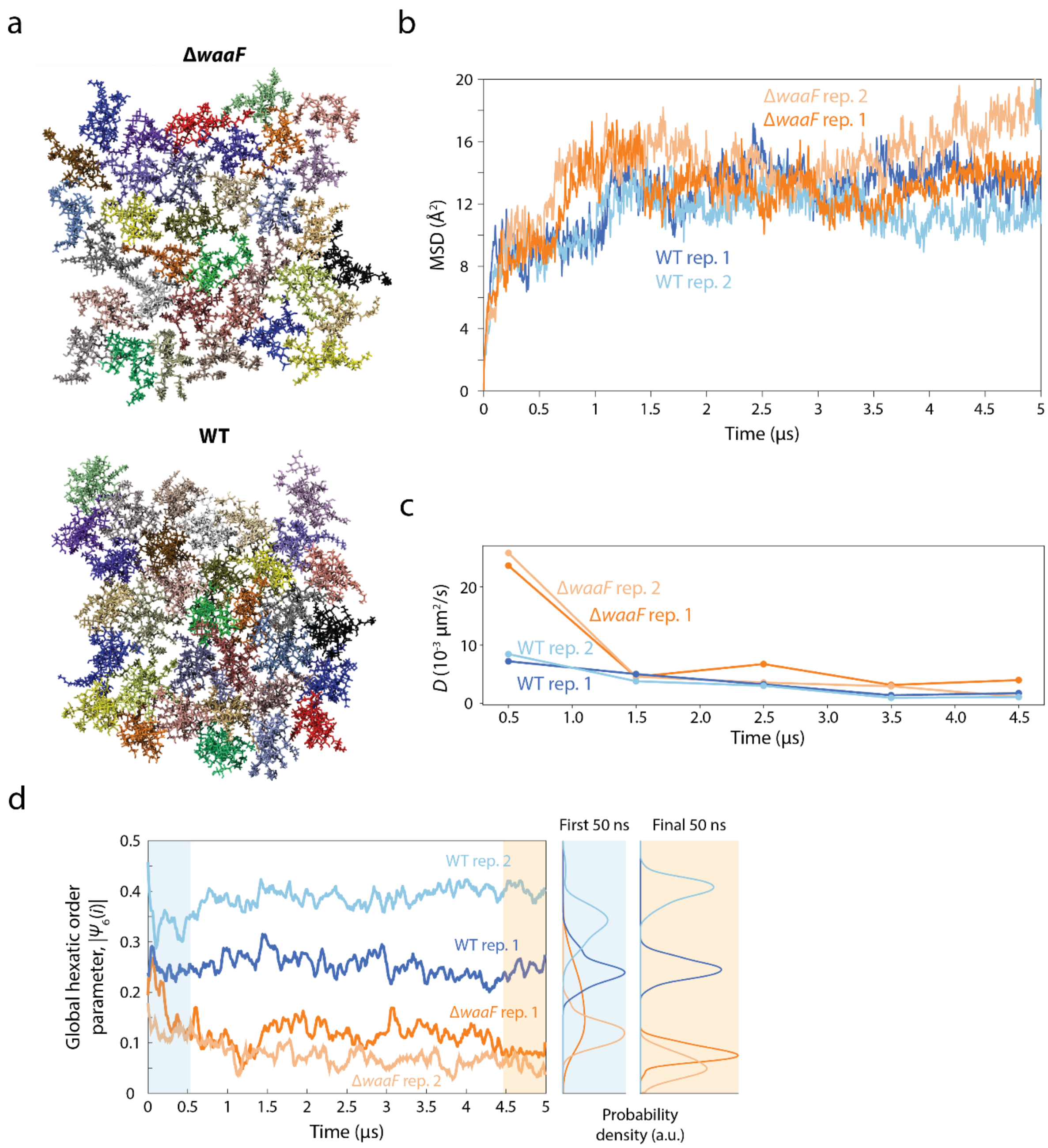

Figure S4: MD simulations yield estimates of LPS diffusion coefficients consistent with experimental measurements.

a) Snapshots of all-atom MD simulations of OM patches containing 34 LPS molecules from wild-type or Δ*waaF* systems (**Methods**).

b) Mean squared displacement (MSD) of LPS molecules during the simulations. Δ*waaF* LPS exhibits a faster initial increase in MSD during the first ~1 μs,

bioRxiv preprint doi: https://doi.org/10.64898/2026.09.17.752130; this version posted September 19, 2026. The copyright holder for this preprint (which was not certified by peer review) is the author/funder, who has granted bioRxiv a license to display the preprint in perpetuity. It is made available under a CC-BY 4.0 International license.

followed by a plateau as molecules become locally confined by neighboring LPS. Because the total simulation time (5 μs) is much shorter than the estimated time (~1 ms) required for an LPS molecule to diffuse across its own lateral dimension, the simulations only capture local motion within a confinement regime.

c) Diffusion coefficients estimated from MSD over 1 μs intervals (D~10^{-3} $\mu m^2/s$) are broadly consistent with values measured by FRAP (D~10^{-3}-10^{-4} $\mu m^2/s$).

d) Wild-type and Δ*waaF* LPS relax into distinct global packing states, quantified by a global hexatic order parameter, $|\Psi_6|$, that reports the global organization of nearest neighbors (**Methods**). Time courses of $|\Psi_6|$ show that truncated LPS adopts a less ordered global packing state than wild-type LPS across independent simulations.

bioRxiv preprint doi: https://doi.org/10.64898/2026.09.17.752130; this version posted September 19, 2026. The copyright holder for this preprint (which was not certified by peer review) is the author/funder, who has granted bioRxiv a license to display the preprint in perpetuity. It is made available under a CC-BY 4.0 International license.

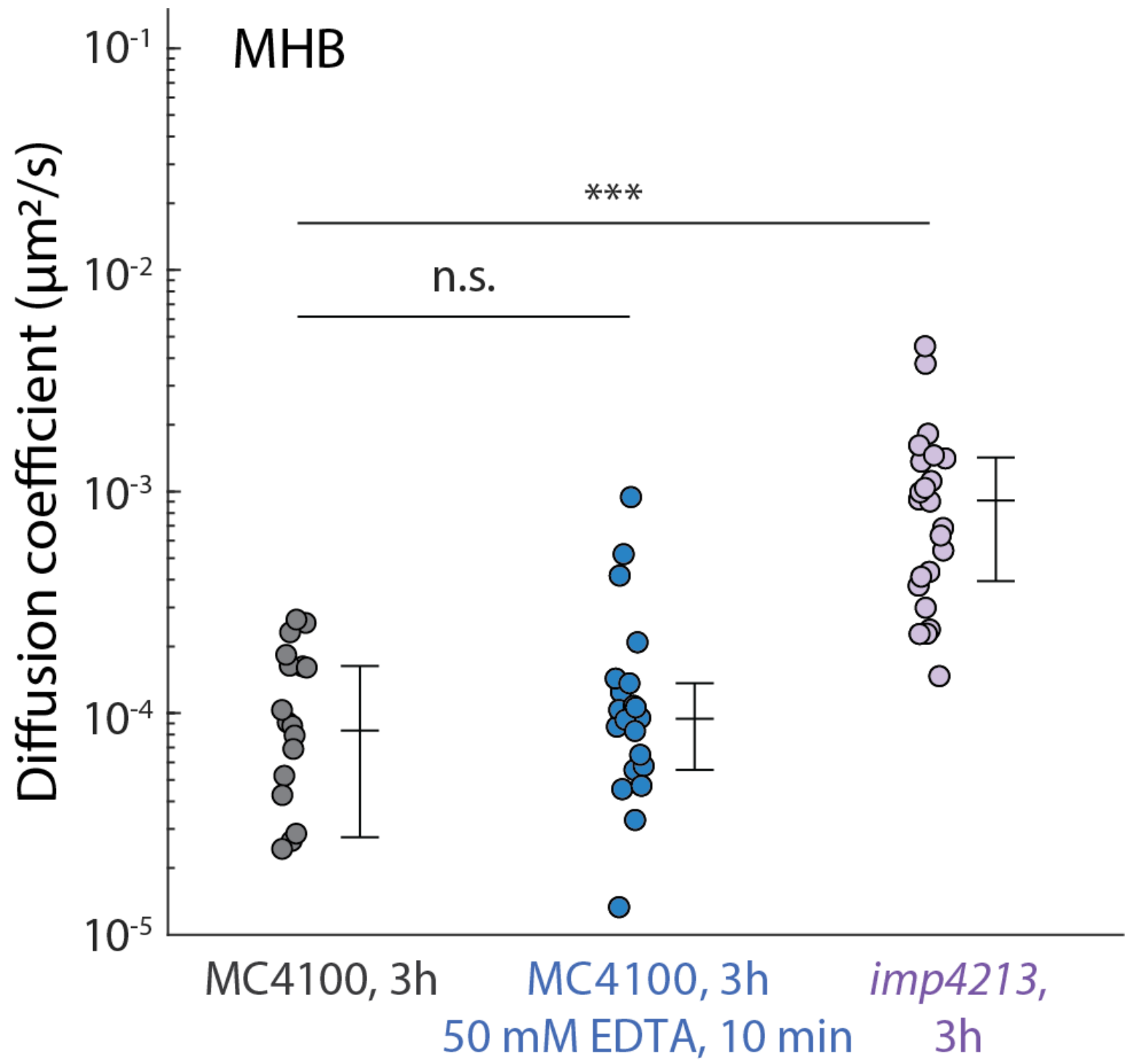

Figure S5: Disrupting LPS crosslinking or reducing LPS levels causes only modest increases in OM fluidity in MHB. Treating wild-type MC4100 cells grown in MHB for 3 h with 50 mM EDTA for 10 min did not significantly increase OM fluidity, consistent with results obtained in LB (**Fig. 4b**). The *imp4213* mutation increased fluidity by ~10-fold in MHB, a larger effect than observed in LB (**Fig. 4a**), but still substantially lower than the fluidization caused by phospholipid accumulation in the outer leaflet (Fig. 1). Center lines indicate medians, and error bars indicate the 25th and 75th percentiles. $N >$ 19 cells per condition. p values were calculated using Welch's t-test on $\log_{10}$-transformed diffusion coefficients. ***: $p < 0.001$; n.s.: $p > 0.05$.

bioRxiv preprint doi: https://doi.org/10.64898/2026.09.17.752130; this version posted September 19, 2026. The copyright holder for this preprint (which was not certified by peer review) is the author/funder, who has granted bioRxiv a license to display the preprint in perpetuity. It is made available under a CC-BY 4.0 International license.

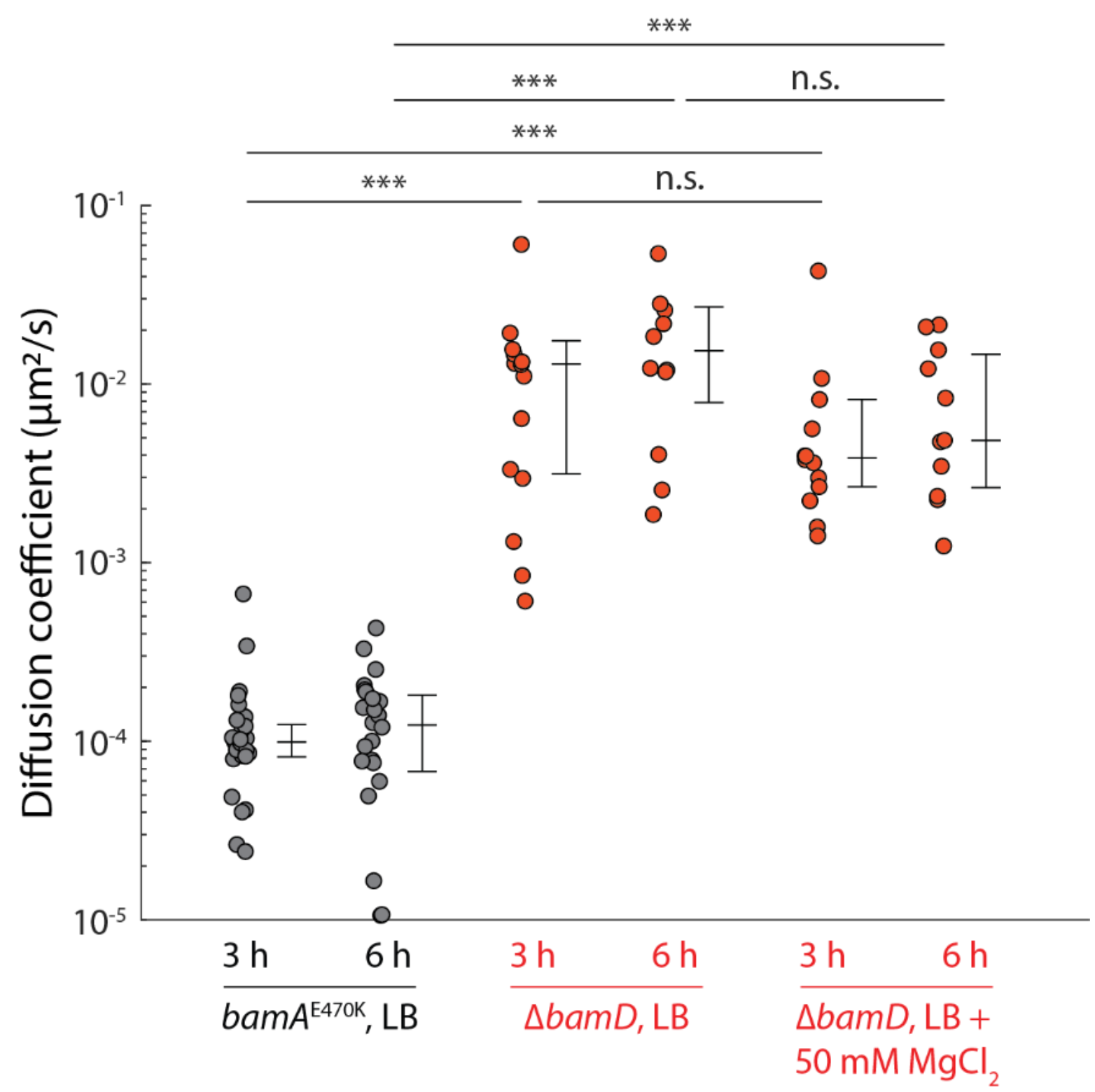

Figure S6: Log-phase Δ*bamD* cells maintain high OM fluidity despite Mg^{2+} supplementation.

Addition of 50 mM $MgCl_2$ to LB did not substantially reduce OM fluidity in Δ*bamD* cells during exponential growth. Cells were grown in LB for 3 h or 6 h prior to measurement. Center lines indicate medians, and error bars indicate the 25th and 75th percentiles. $N > 10$ cells per condition. p values were calculated using Welch's t-test on $\log_{10}$-transformed diffusion coefficients. ***: $p < 0.001$; n.s.: $p > 0.05$.

bioRxiv preprint doi: https://doi.org/10.64898/2026.09.17.752130; this version posted September 19, 2026. The copyright holder for this preprint (which was not certified by peer review) is the author/funder, who has granted bioRxiv a license to display the preprint in perpetuity. It is made available under a CC-BY 4.0 International license.

Supplementary Movies

Movie S1: Visualization of LPS interaction energies during an MD simulation of an *E. coli* OM patch of wild-type LPS (replicate 1). The movie shows the nonbonded interaction energy between each of the 34 individual LPS molecules and their nearest neighbors. In the calculations, each LPS molecule was treated as a separate unit and included divalent cations (Mg^{2+}, Ca^{2+}) within 4 Å to account for their role in stabilizing intermolecular interactions.

Movie S2: Visualization of LPS interaction energies during an MD simulation of an *E. coli* OM patch of wild-type LPS (replicate 2). Visualization and interaction-energy calculations are as described for Movie S1.

Movie S3: Visualization of LPS interaction energies during an MD simulation of an *E. coli* OM patch of Δ*waaF* LPS (replicate 1). Visualization and interaction-energy calculations are as described for Movie S1.

Movie S4: Visualization of LPS interaction energies during an MD simulation of an *E. coli* OM patch of Δ*waaF* LPS (replicate 2). Visualization and interaction-energy calculations are as described for Movie S1.

Movie S5: Time evolution of inter-LPS interaction networks during an MD simulation of an *E. coli* OM patch of wild-type LPS (replicate 1). Each LPS molecule is represented by a sphere at its center of mass, with cylinders connecting interacting pairs. Cylinder thickness is proportional to the magnitude of the pairwise nonbonded interaction energy (scaled by 0.001), while blue and red indicate favorable (negative) and unfavorable (positive) interactions. The central simulation cell and its eight

bioRxiv preprint doi: https://doi.org/10.64898/2026.09.17.752130; this version posted September 19, 2026. The copyright holder for this preprint (which was not certified by peer review) is the author/funder, who has granted bioRxiv a license to display the preprint in perpetuity. It is made available under a CC-BY 4.0 International license.

neighboring periodic images are shown to visualize interactions across periodic boundaries; non-hydrogen atoms of LPS molecules are shown in the central cell for structural context. Pairwise interaction energies were calculated using the `namdenergy` plugin with periodic electrostatics, with each LPS selection including Ca^{2+}/Mg^{2+} ions within 4 Å.

Movie S6: Time evolution of inter-LPS interaction networks during an MD simulation of an *E. coli* OM patch of wild-type LPS (replicate 2). Visualization and interaction-energy calculations are as described for Movie S5.

Movie S7: Time evolution of inter-LPS interaction networks during an MD simulation of an *E. coli* OM patch of Δ*waaF* LPS (replicate 1). Visualization and interaction-energy calculations are as described for Movie S5.

Movie S8: Time evolution of inter-LPS interaction networks during an MD simulation of an *E. coli* OM patch of Δ*waaF* LPS (replicate 2). Visualization and interaction-energy calculations are as described for Movie S5.

bioRxiv preprint doi: https://doi.org/10.64898/2026.09.17.752130; this version posted September 19, 2026. The copyright holder for this preprint (which was not certified by peer review) is the author/funder, who has granted bioRxiv a license to display the preprint in perpetuity. It is made available under a CC-BY 4.0 International license.

Supplementary Table

Strain	Genotype	Source/reference
KC671	MC4100 *ara+*	[78]
IMB1047	MC4100 *ara^R bamA^{E470K}*	[16]
IMB1056	MC4100 *ara^R bamA^{E470K}* Δ*bamD*	[16]
IMB1132	MC4100 *ara^R bamA^{E470K}* Δ*bamD* Δ*pldA* Δ*mlaA*	[16]
HC726	MC4100 *ara$^+$* Δ*yfdI mlaA* pldA*::*kan*	[46]
BH92	MC4100 *ara^R bamB*::*kan*	[16]
BW25113	Δ(*araD-araB*)*567* Δ(*rhaD-rhaB*)*568* Δ*lacZ4787* (::rrnB-3) *hsdR514 rph-1*	Gift from Jie Xiao
Δ*waaD*	BW25113 Δ*waaD*::*kan*	[51]
Δ*waaF*	BW25113 Δ*waaF*::*kan*	[51]
Δ*waaG*	BW25113 Δ*waaG*::*kan*	[51]
Δ*waaP*	BW25113 Δ*waaP*::*kan*	[51]
Δ*waaJ*	BW25113 Δ*waaJ*::*kan*	[51]
Δ*waaY*	BW25113 Δ*waaY*::*kan*	[51]
lptC CRISPRi	SJ_XTL219 *lptC* sgRNA	[66]
lpxC CRISPRi	SJ_XTL219 *lpxC* sgRNA	[66]
bamD CRISPRi	BW25113 Tn7att::Tn7C59, λatt::sgRNA(*bamD*)	[67]
imp4213	MC4100 *imp4213 carB*::Tn10	[44]

Table S1: *E. coli* strains used in this study.
